# Prognostic value of histopathological DCIS features in a large-scale international interrater reliability study

**DOI:** 10.1007/s10549-020-05816-x

**Published:** 2020-07-30

**Authors:** Emma J. Groen, Jan Hudecek, Lennart Mulder, Maartje van Seijen, Mathilde M. Almekinders, Stoyan Alexov, Anikó Kovács, Ales Ryska, Zsuzsanna Varga, Francisco-Javier Andreu Navarro, Simonetta Bianchi, Willem Vreuls, Eva Balslev, Max V. Boot, Janina Kulka, Ewa Chmielik, Ellis Barbé, Mathilda J. de Rooij, Winand Vos, Andrea Farkas, Natalja E. Leeuwis-Fedorovich, Peter Regitnig, Pieter J. Westenend, Loes F. S. Kooreman, Cecily Quinn, Giuseppe Floris, Gábor Cserni, Paul J. van Diest, Esther H. Lips, Michael Schaapveld, Jelle Wesseling, Grand Challenge PRECISION consortium

**Affiliations:** 1 Department of Pathology, Netherlands Cancer Institute – Antoni van Leeuwenhoek, Plesmanlaan 121, 1066 CX Amsterdam, The Netherlands; 2 Department of Research IT, Netherlands Cancer Institute – Antoni van Leeuwenhoek, Amsterdam, The Netherlands; 3 Department of Molecular Pathology, Netherlands Cancer Institute – Antoni van Leeuwenhoek, Amsterdam, The Netherlands; 4 Department of Pathology, Oncology Hospital, Sofia, Bulgaria; 5 Department of Clinical Pathology, Sahlgrenska University Hospital, Gothenburg, Sweden; 6 The Fingerland Department of Pathology, Charles University Medical Faculty and University Hospital Hradec Kralove, Hradec Kralove, Czech Republic; 7 Institute of Pathology and Molecular Pathology, University Hospital Zurich, Zurich, Switzerland; 8 Atryshealth Co, S.L., Barcelona, Spain; 9 Division of Pathological Anatomy, Department of Health Sciences, University of Florence, Florence, Italy; 10 Department of Pathology, Canisius Wilhelmina Hospital, Nijmegen, The Netherlands; 11 Department of Pathology, Herlev University Hospital, Herlev, Denmark; 12 Department of Pathology, Amsterdam University Medical Center, Location VUmc, Amsterdam, The Netherlands; 13 2nd Department of Pathology, Semmelweis University, Budapest, Hungary; 14 Tumor Pathology Department, Maria Sklodowska-Curie National Research Institute of Oncology, Gliwice Branch, Gliwice, Poland; 15 Symbiant Pathology Expert Centre, Location ZMC, Zaandam, The Netherlands; 16 Department of Pathology, Zuyderland Medical Center, Location Sittard-Geleen, Sittard-Geleen, The Netherlands; 17 Department of Pathology, Gävle Hospital, Gävle, Sweden; 18 Department of Pathology, Deventer Hospital, Deventer, The Netherlands; 19 Diagnostic and Research Institute of Pathology, Medical University of Graz, Graz, Austria; 20 Laboratory for Pathology Dordrecht, Dordrecht, The Netherlands; 21 Department of Pathology and GROW School for Oncology and Developmental Biology, Maastricht University Medical Centre, Maastricht, The Netherlands; 22 Department of Pathology and Laboratory Medicine, St. Vincent’s University Hospital, Dublin, Ireland; 23 Laboratory of Translational Cell & Tissue Research, Department of Imaging and Pathology, KU Leuven - University of Leuven, Leuven, Belgium; 24 Department of Pathology, University Hospitals Leuven, Leuven, Belgium; 25 Department of Pathology, Bács-Kiskun County Teaching Hospital, Kecskemét, Hungary; 26 Department of Pathology, University of Szeged, Szeged, Hungary; 27 Department of Pathology, University Medical Center Utrecht, Utrecht, The Netherlands; 28 Division of Psychosocial Research and Epidemiology, Netherlands Cancer Institute – Antoni van Leeuwenhoek, Amsterdam, The Netherlands

**Keywords:** Ductal carcinoma in situ, Invasive breast cancer, Interrater reliability, Risk stratification

## Abstract

**Purpose:**

For optimal management of ductal carcinoma in situ (DCIS), reproducible histopathological assessment is essential to distinguish low-risk from high-risk DCIS. Therefore, we analyzed interrater reliability of histopathological DCIS features and assessed their associations with subsequent ipsilateral invasive breast cancer (iIBC) risk.

**Methods:**

Using a case-cohort design, reliability was assessed in a population-based, nationwide cohort of 2767 women with screen-detected DCIS diagnosed between 1993 and 2004, treated by breast-conserving surgery with/without radiotherapy (BCS ± RT) using Krippendorff’s alpha (KA) and Gwet’s AC2 (GAC2). Thirty-eight raters scored histopathological DCIS features including grade (2-tiered and 3-tiered), growth pattern, mitotic activity, periductal fibrosis, and lymphocytic infiltrate in 342 women. Using majority opinion-based scores for each feature, their association with subsequent iIBC risk was assessed using Cox regression.

**Results:**

Interrater reliability of grade using various classifications was fair to moderate, and only substantial for grade 1 versus 2 + 3 when using GAC2 (0.78). Reliability for growth pattern (KA 0.44, GAC2 0.78), calcifications (KA 0.49, GAC2 0.70) and necrosis (KA 0.47, GAC2 0.70) was moderate using KA and substantial using GAC2; for (type of) periductal fibrosis and lymphocytic infiltrate fair to moderate estimates were found and for mitotic activity reliability was substantial using GAC2 (0.70). Only in patients treated with BCS-RT, high mitotic activity was associated with a higher iIBC risk in univariable analysis (Hazard Ratio (HR) 2.53, 95% Confidence Interval (95% CI) 1.05–6.11); grade 3 versus 1 + 2 (HR 2.64, 95% CI 1.35–5.14) and a cribriform/solid versus flat epithelial atypia/clinging/(micro)papillary growth pattern (HR 3.70, 95% CI 1.34–10.23) were independently associated with a higher iIBC risk.

**Conclusions:**

Using majority opinion-based scores, DCIS grade, growth pattern, and mitotic activity are associated with iIBC risk in patients treated with BCS-RT, but interrater variability is substantial. Semi-quantitative grading, incorporating and separately evaluating nuclear pleomorphism, growth pattern, and mitotic activity, may improve the reliability and prognostic value of these features.

**Electronic supplementary material:**

The online version of this article (10.1007/s10549-020-05816-x) contains supplementary material, which is available to authorized users.

## Background

Ductal carcinoma in situ (DCIS) of the breast is a non-obligate precursor of invasive breast cancer (IBC). Since the introduction of organized population-based breast screening, the incidence of DCIS has increased manyfold [1–3]. Although DCIS is almost always treated to avoid progression to IBC, this has not led to a reduced IBC incidence. Breast screening programs are therefore criticized by some for being associated with overdiagnosis and overtreatment of DCIS [4–6]. It has been reported that a large proportion of untreated DCIS will not progress to IBC [7, 8]. Ryser et al. reported a 10-year net risk of ipsilateral IBC (iIBC) of 12.2% (95% Confidence Interval (95% CI) 8.6–17.1%) for women with DCIS grade 1/2 and 17.6% (95% CI 12.1–25.2%) for grade 3 [8]. Although based on selected patients, these results underline that at least some DCIS lesions have a low risk of progression and may thus be overtreated. However, reliably distinguishing high- from low-risk DCIS to guide treatment is still challenging.

Many studies have tried to find histopathological markers that could predict progression of DCIS [9, 10]. So far, no single marker ended up being used in clinical practice due to lack of conclusive evidence of predictive ability, in part due to suboptimal biased study designs in particular due to insufficient handling of confounders and poorly described study groups [10]. Especially grade has been extensively studied as a biomarker for the invasive potential of DCIS. The use of many different grading systems with partly unclear criteria and often only poor to modest interrater reliability makes it difficult to evaluate the role of grade in risk stratification [11–21].

In addition, various studies have assessed reproducibility of histopathological evaluation of DCIS lesions. Unfortunately, these studies were frequently based on highly selected case sets, assessed by expert breast pathologists often after having received instructions or tutorials beforehand and using reference diagnoses without follow-up data [17, 18, 22–28]. The interpretation of results and evaluation of potential bias is further complicated by inadequate reporting [29].

This study assesses the interrater reliability of various histopathological features in DCIS in a setting which as closely as possible reflects daily practice. We subsequently evaluate whether these features, based on a more robust majority opinion of 38 raters, are associated with risk of development of subsequent iIBC.

## Methods

### Patient selection

We assembled a population-based, nationwide cohort of screen-detected primary and pure DCIS, treated with breast-conserving surgery with or without adjuvant radiotherapy (BCS ± RT) between January 1, 1993 and December 31, 2004, by linkage of data from the Netherlands Cancer Registry (NCR) with data from the Dutch breast cancer screening program [30]. From 1989, the Dutch biennial screening program was gradually introduced, inviting women aged 50–69 years and from 1998 aged 50–75 years. Screen-detected DCIS was defined as DCIS detected within 30 months after a first or subsequent positive screening examination. The cohort was supplemented with data from the nationwide network and registry of histology and cytopathology in the Netherlands (PALGA) [31]. Information on age and date at diagnosis, treatment, and if applicable subsequent iIBC and vital status was provided by the NCR (follow-up data available until January 1, 2011). Patients diagnosed with a prior malignancy, other than non-melanoma skin cancer, were excluded. The review boards of the NCR, PALGA and the Dutch breast cancer screening organization approved this study.

### Interrater reliability analysis

**Fig. 1:**
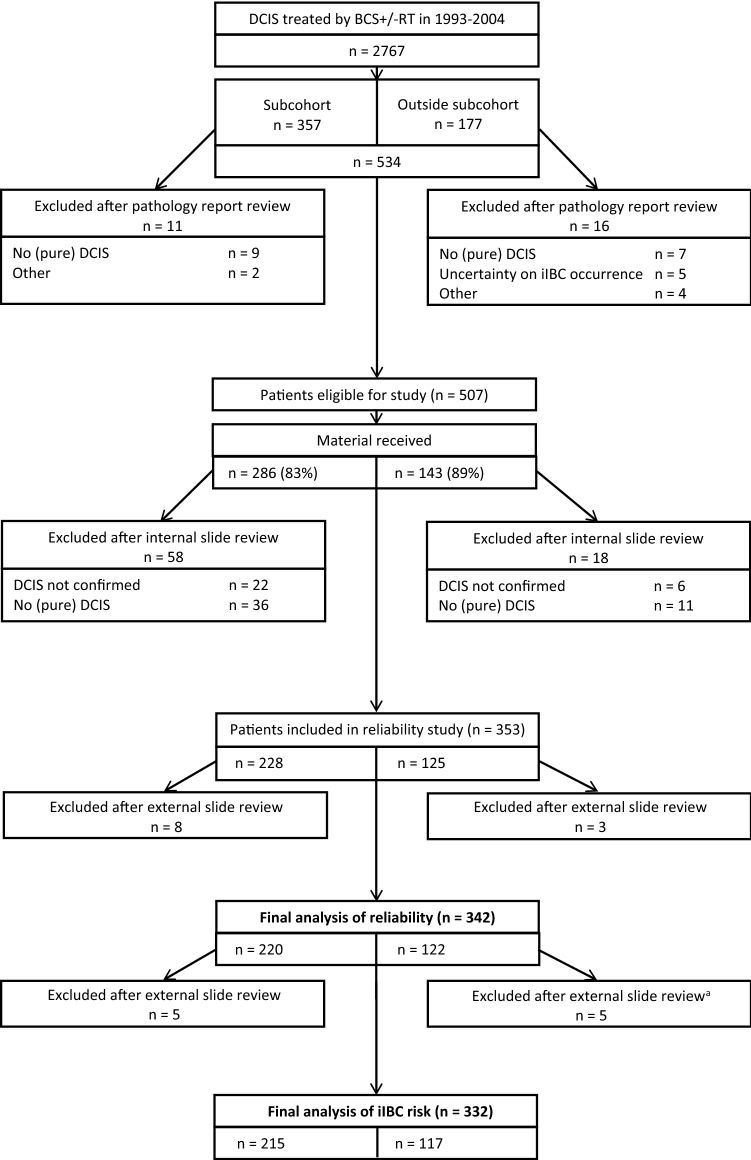
Flow diagram for patient selection and exclusions *Subcohort* randomly selected patient group; *outside* *subcohort* patients who developed subsequent ipsilateral invasive breast cancer not included in the subcohort, *iIBC* ipsilateral invasive breast cancer; ^a^2 outside subcohort patients developed invasive breast cancer after a mastectomy was performed during follow-up, for other reasons than iIBC.

We first assessed the interrater reliability of histopathological DCIS features in this cohort using a case-cohort design [32]. From the cohort of 2767 women, we randomly sampled 357 women (subcohort; 13%) and additionally selected all 177 patients who subsequently developed an iIBC but were not included in the random sample for a total of 534 patients. Figure 1 shows the selection of patients with exclusions at pathology report review (*n* = 27) and slide review (*n* = 76). Slide review was based on freshly cut slides stained with hematoxylin and eosin and in case of uncertainty about the in situ nature of the lesion also with cytokeratin 14 by EJG (clone LL002; 1/3200 dilution, 32 min at 37 °C + amplification, Neomarkers/Thermo Scientific).

For 353 patients the diagnosis of pure DCIS could be confirmed and from each lesion a single slide was selected with the highest quantity of DCIS. These slides were digitized using an Aperio AT2 scanner (Leica Biosystems) at 20 × magnification and uploaded on an online viewing platform (https://www.slidescore.com/). For each DCIS lesion a scoring form (see Supplementary methods) was built-in with the items: DCIS present (yes or no), grade (1, 2, or 3), grade (low or high), growth pattern (flat epithelial atypia (FEA), clinging, (micro)papillary, cribriform, or solid) and mitotic activity of DCIS (sparse or many mitoses), calcifications (present or absent), necrosis (present or absent), periductal fibrosis (absent, subtle, or prominent) and lymphocytic infiltrate (absent, subtle, or prominent). For each item, a ‘not assessable’ category was also provided. Regarding DCIS growth patterns, there is controversy about whether to consider FEA as a subtype of DCIS (clinging, monomorphic type) or not; therefore, this option was included as possible DCIS growth pattern.

European raters with varying expertise were invited to participate in the study. Each rater was assigned a study set of 146 cases to score independently, blinded to subject information. Raters were not given instructions regarding the (interpretation of) histopathological features and were requested to score as they would in daily practice to provide an unbiased baseline measure of reliability. Further details on rater selection, participation, and the scoring process are described in Supplementary methods.

### Statistical analysis

In total, 11 patients were excluded from reliability analysis because > 50% of raters considered their lesion as no DCIS/not assessable (often considering atypical ductal hyperplasia/FEA as alternative diagnosis; *n* = 5) or > 25% commented on suboptimal slide quality (*n* = 6). If DCIS was not confirmed, any scores for following histopathological features were ignored. Scores for type of fibrosis were only considered when periductal fibrosis was present according to the majority opinion. Raters were excluded for the analysis of single histopathological features when they scored an item as ‘not assessable’ in > 50% of their study set.

Krippendorff’s alpha (KA), Gwet’s AC2 (GAC2), and percentage agreement were calculated to assess interrater reliability (‘not assessable’ scores were excluded) [33, 34]. KA and GAC2 are applicable to studies involving nominal/ordinal data and multiple raters scoring different subsets. A weighted analysis using linear weights was used for ordinal variables with > 2 categories. Interpretation was performed according to Landis and Koch [35]. Recategorization of grade, periductal fibrosis, and lymphocytic infiltrate was undertaken during analysis to evaluate reliability using different cut-offs.

For the analysis of subsequent iIBC risk, an additional 10 patients were excluded, because > 25% of the raters considered an invasive carcinoma component (mainly microinvasion) to be present adjacent to DCIS (*n* = 8) or because the patient underwent a mastectomy before developing iIBC (*n* = 2). For a detailed comparison of clinical characteristics between in- versus excluded patients see Supplementary Table S1.

Associations of histopathological features, treatment, age at diagnosis, and period of diagnosis (1993–1998, reflecting the screening implementation phase, versus 1999–2004, reflecting full nationwide coverage) with risk of iIBC was assessed using Cox models. Analyses were performed irrespective of treatment as well as separately for BCS alone and BCS + RT. Interactions with treatment were also considered. Proportional hazard assumptions (PHA) were tested using residual-based and graphical methods. In case the PHA was violated, a time factor was added, and the associations were estimated for different time-periods (i.e., for the first 5 years and after 5 years). For the histopathological features the majority opinion, i.e., the most frequently assigned category, was used in the analysis (‘not assessable’ scores were excluded). In case of equal frequencies, the presence of a histopathological feature was chosen over absence, the highest grade, the most complex growth pattern (i.e., cribriform/solid), many over sparse mitoses, prominent over subtle presence for periductal fibrosis and lymphocytic infiltrate and the least common type of fibrosis (i.e., myxoid). Time to iIBC was compared between women with low-grade DCIS versus high-grade DCIS and women treated with BCS + RT versus BCS alone using median test. Clinicopathological factors were entered in multivariable models including treatment, based on a *P* value ≤ 0.15 in univariable analyses. Barlow’s inverse probability weights were used to adjust the partial likelihood function for case-cohort analysis with robust variance estimation [32]. Fit of non-nested models was compared using Akaike's and Bayesian information criteria. Two-sided *P* values ≤ 0.05 were considered statistically significant. All statistical analyses were performed using Stata/SE (version 13.1, Statacorp).

## Results

### Interrater reliability

The mean number of scores per slide was 14 (range 12–15) (Supplementary Table S2). The raters consisted of a mixed group (Supplementary Table S3), about half of them working in the Netherlands and half in other European countries within a wide range of laboratories regarding size and degree of specialization. Forty-seven percent of raters were members of the European Working Group of Breast Screening Pathologists. The diagnosis of DCIS was confirmed in 98.6% of the patients based on the majority opinion.

The interrater reliability for the 3-tiered grading system (grade 1, 2, or 3), the most commonly used histopathological feature, was only fair (KA 0.34; 95% CI 0.30–0.39) to moderate (GAC2 0.52; 95% CI 0.50–0.55; Table 1). Using a 2-tiered grading system (either low versus high grade or grade 1 + 2 versus grade 3) did not improve reliability. When the 3-tiered grading was recategorized into a category for grade 1 and a category for grade 2 + 3 combined, the reliability was substantial using GAC2 (0.78; 95% CI 0.74–0.82).

**Table 1:** Agreement, Gwet’s AC2 (GAC2), and Krippendorff’s alpha (KA) coefficients per histopathological feature

Histopathological feature	Agreement (%)	95% CI (%)	GAC2	95% CI	KA	95% CI
Grade (1, 2 or 3)	76.4	75.27–77.52	0.52	0.50–0.55	0.34	0.30–0.39
Grade (1 versus 2 + 3)	83.5	81.33–85.68	0.78	0.74–0.82	0.35	0.28–0.42
Grade (1 + 2 versus 3)	69.3	66.94–71.63	0.43	0.38–0.49	0.34	0.29–0.38
Grade (low versus high)	72.8	70.54–75.12	0.52	0.47–0.57	0.38	0.32–0.44
Dominant growth pattern	84.8	82.58–86.97	0.78	0.75–0.82	0.44	0.37–0.51
Calcifications	81.1	78.81–83.40	0.70	0.65–0.75	0.49	0.43–0.54
Necrosis	81.4	79.12–83.64	0.70	0.66–0.75	0.47	0.41–0.53
Mitotic activity	78.5	76.12–80.97	0.70	0.65–0.74	0.24	0.19–0.29
Periductal fibrosis (absent, subtle or prominent presence)	70.9	69.71–72.13	0.37	0.34–0.39	0.25	0.22–0.29
Periductal fibrosis (present versus absent)	71.2	68.82–73.48	0.53	0.48–0.58	0.23	0.18–0.28
Type of periductal fibrosis (if present)	70.5	67.57–73.37	0.50	0.44–0.57	0.26	0.21–0.31
Lymphocytic infiltrate (absent, subtle or prominent presence)	77.1	75.82–78.36	0.50	0.47–0.53	0.42	0.38–0.47
Lymphocytic infiltrate (present versus absent)	73.0	70.51–75.40	0.51	0.45–0.56	0.38	0.33–0.43

*GAC2* Gwet’s AC2, *KA* Krippendorff’s alpha, weighted analysis was performed for ordinal features with more than 2 categories using linear weights (grade 1–3, periductal fibrosis, and lymphocytic infiltrate), *CI* confidence interval

Comparable moderate (KA) to substantial (GAC2) reliability was found for growth pattern, necrosis, and calcifications, which are all features assessed in daily practice within the context of DCIS. FEA was scored 38 times in 24 different patients (representing 0.76% of all evaluations); in only 1 patient FEA was the majority opinion. Reliability did not change when FEA scores were excluded from analysis. A striking discrepancy in reliability was found for the assessment of mitotic activity with only fair reliability when considering KA (0.24) but substantial reliability based on GAC2 (0.70). In a 3-tiered system (absent, subtle, or prominent presence), lymphocytic infiltrate showed moderate reliability, which was slightly better than the interrater reliability for periductal fibrosis. Recategorization, comparing periductal fibrosis presence with absence led to a moderate reliability (GAC2 0.53).

### Risk of subsequent iIBC after DCIS

**Fig. 2:**
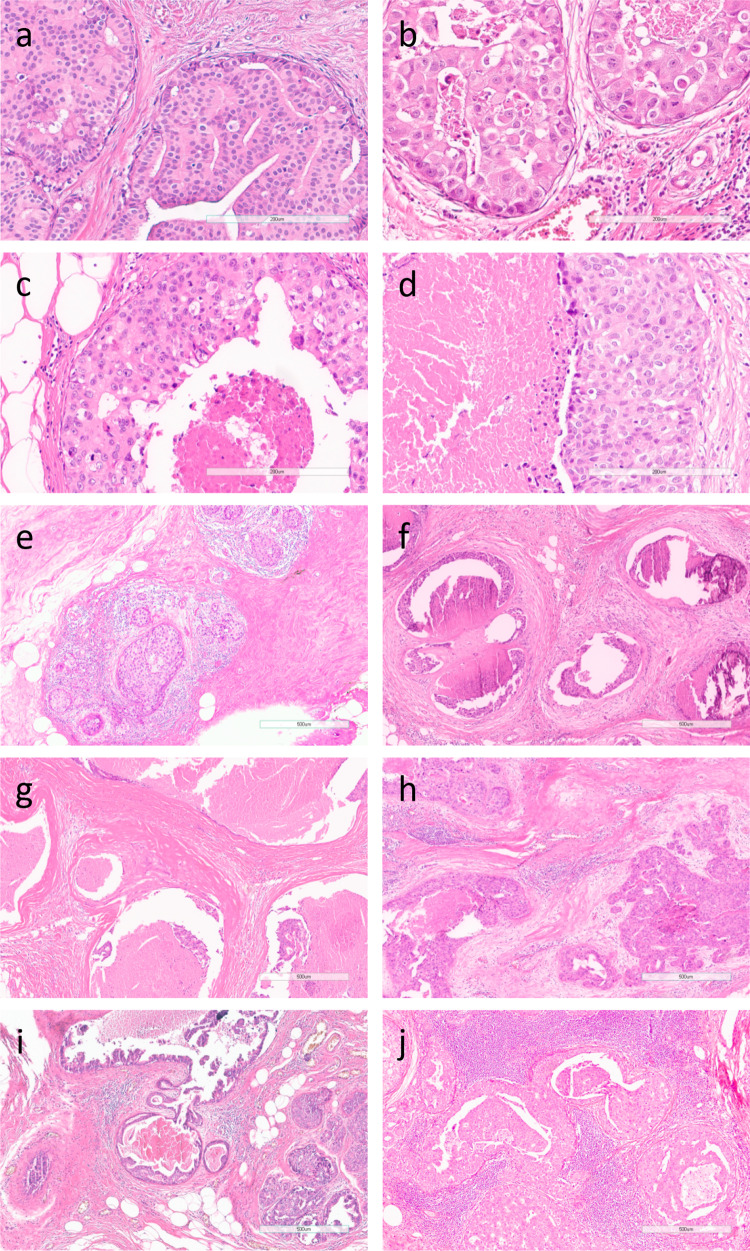
Photomicrographs from histopathological DCIS features based on the majority opinion. **a** low-grade DCIS (hematoxylin and eosin (H&E); × 200), **b** high-grade DCIS (H&E; × 200), **c** many mitoses (H&E; × 200), **d** necrosis (H&E; × 200), **e** subtle periductal fibrosis (H&E; × 50), **f** prominent periductal fibrosis (H&E; × 50), **g** sclerotic periductal fibrosis (H&E; × 50), **h** myxoid periductal fibrosis (H&E; × 50), **i** subtle periductal lymphocytic infiltrate (H&E; × 50), **j** prominent periductal lymphocytic infiltrate (H&E; × 50)

Subcohort patients were diagnosed with DCIS at a median age of 58.4 (interquartile range 53.4–64.0) and treated by BCS alone in 40.5% (87 patients) and by BCS + RT in 59.5% (128 patients). After a median follow-up of 11.2 years (interquartile range 8.6–14.1), 20 patients developed an iIBC in the subcohort. DCIS was assigned grade 1 in 10.7%, grade 2 in 53.5%, and grade 3 in 35.8%, based on the majority opinion. Median time to iIBC was 5.3 years (interquartile range 3.3–7.6 years). Time to subsequent iIBC for women with low-grade DCIS did not differ significantly from those with high-grade DCIS (median 5.3 years versus 5.6 years, respectively, *P* = 0.57). Time to iIBC for women treated with BCS + RT (median 5.9 years) did also not differ significantly from those treated with BCS alone (median 5.1 years); *P* = 0.12). Table 2 shows clinicopathological characteristics of the subcohort and of all patients who developed an iIBC and Fig. 2 depicts photomicrographs of several histopathological DCIS features based on the majority opinion.

**Table 2:** Clinical characteristics and histopathological characteristics (based on the majority opinion) of the study population

Number of DCIS patients (%)	All patients with iIBC 137*	Subcohort 215**
Treatment		
BCS + RT	42 (30.7)	128 (59.5)
BCS alone	95 (69.3)	87 (40.5)
Age at DCIS diagnosis, years, median (iqr)	57.5 (53.1–63.6)	58.4 (53.4–64.0)
Age at DCIS diagnosis, years (quartiles)		
≥ 49.5—≤ 53.4	37 (27.0)	54 (25.1)
> 53.4—≤ 58.2	36 (26.3)	50 (23.3)
> 58.2—≤ 63.8	32 (23.4)	56 (26.1)
> 63.8—≤ 75.6	32 (23.4)	55 (25.6)
Period of DCIS diagnosis^a^		
1993—1998	76 (55.5)	82 (38.1)
1999—2004	61 (44.5)	133 (61.9)
Median follow-up, years (iqr)		11.2 (8.6–14.1)
Time to iIBC, years, median (iqr)	5.3 (3.3–7.6)	
Grade (1,2 or 3)		
Grade 1	10 (7.3)	23 (10.7)
Grade 2	67 (48.9)	115 (53.5)
Grade 3	60 (43.8)	77 (35.8)
Grade (low versus high)		
Low grade	31 (22.6)	60 (27.9)
High grade	106 (77.4)	155 (72.1)
Dominant growth pattern^b^		
FEA, clinging, (micro)papillary	14 (10.2)	34 (15.9)
Cribriform, solid	123 (89.8)	180 (84.1)
Calcifications		
Present	103 (75.2)	168 (78.1)
Absent	34 (24.8)	47 (21.9)
Necrosis		
Present	109 (79.6)	167 (77.7)
Absent	28 (20.4)	48 (22.3)
Mitoses		
Sparse	114 (83.2)	198 (92.1)
Many	23 (16.8)	17 (7.9)
Periductal fibrosis		
Absent	28 (20.4)	41 (19.1)
Subtle	73 (53.4)	102 (47.4)
Prominent	36 (26.3)	72 (33.5)
Type of periductal fibrosis^c^		
Sclerotic	80 (73.4)	133 (76.4)
Myxoid	29 (26.6)	41 (23.6)
Lymphocytic infiltrate		
Absent	38 (27.7)	77 (35.8)
Subtle	65 (47.5)	89 (41.4)
Prominent	34 (24.8)	49 (22.8)

*subcohort* randomly selected patient group, *iqr* interquartile range

^*^Six out of all patients with iIBC developed breast cancer metastases only

**Sixteen patients from the subcohort developed an iIBC and four developed breast cancer metastases only

^a^1993–1998 reflecting part of the screening implementation phase and 1999–2004 reflecting full nationwide coverage

^b^In one patient, growth pattern was scored as not assessable by all raters and was therefore excluded (n included patients = 331); FEA = flat epithelial atypia

^c^For type of fibrosis, patients were only included when according to the majority opinion periductal fibrosis was present, either subtle or prominent (n included patients = 268)

In univariable analysis, patients treated with BCS alone had a much higher risk of iIBC than patients treated with BCS + RT with a Hazard Ratio (HR) of 4.80 (95% CI 2.49–9.24) in the first 5 years and a HR of 2.47 after 5 years (95% CI 1.42–4.30; Supplementary Table S4). In patients treated with BCS alone, grade 3 (versus grade 1 + 2 combined), a cribriform/solid growth pattern (versus FEA, clinging, and (micro)papillary growth pattern), and mitotically active DCIS (versus DCIS with low mitotic activity) were also associated with a higher iIBC risk, whereas in patients treated with BCS + RT these associations were not found. In univariable analysis, a significant interaction with treatment was found for grade 3 versus 1 + 2 (*P* = 0.028) and for growth pattern (*P* = 0.023).

**Fig. 3:**
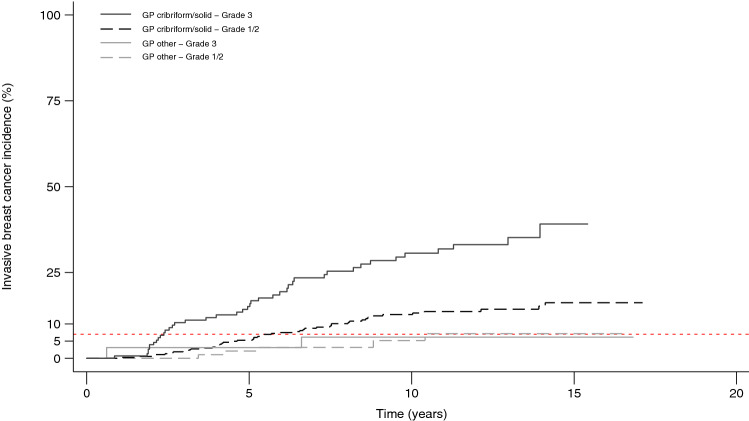
Kaplan–Meier curve illustrating iIBC incidence after diagnosis of DCIS treated by BCS alone. *GP* growth pattern, *other* flat epithelial atypia, clinging and (micro)papillary growth pattern. The red dashed reference line depicts the maximum reached incidence in patients with DCIS grade 3 with a cribriform/solid growth pattern treated with BCS + RT

In multivariable analysis, a model which, besides treatment, included grade 3 versus grade 1 + 2 and growth pattern (cribriform and solid versus FEA, clinging, and (micro)papillary) best predicted the risk of developing iIBC in patients treated with BCS alone, while grade and growth pattern were not associated with iIBC risk in patients treated with BCS + RT (Table 3). The risk of developing iIBC did not differ between patients with DCIS grade 1/2 and FEA, clinging, or (micro)papillary growth pattern who were treated with BCS alone or BCS + RT. Figure 3 shows cumulative risk of iIBC based on categories derived from this model.

**Table 3:** Associations of histopathological features with subsequent iIBC in multivariable analysis

Histopathological feature	BCS alone			BCS + RT			Treatment interaction
	*n*	HR (95% CI)	*P*	*n*	HR (95% CI)	*P*	*P*
Grade (1 + 2 versus 3)							0.017
1 + 2	107 (52)	REF		104 (28)	REF		
3	62 (43)	2.64 (1.35–5.14)	0.005	58 (14)	0.79 (0.38–1.62)	0.52	
Dominant growth pattern							0.022
FEA/clinging/(micro)papillary	23 (7)	REF		23 (7)	REF		
Cribriform/solid	146 (88)	3.70 (1.34–10.23)	0.012	139 (35)	0.77 (0.32–1.85)	0.56	

*n* total number (number of patients with subsequent iIBC), *HR* Hazard Ratio, *CI* confidence interval, *P P* value, *REF* reference, *FEA* flat epithelial atypia

## Discussion

To the best of our knowledge, this is the first study combining a comprehensive interrater reliability study in DCIS, reflecting daily practice as closely as possible, with an analysis of iIBC risk based on the majority opinion of a large group of raters. This approach minimizes the muddling effect of interrater variability and subjectivity on the evaluation of the prognostic value of histopathological features. It will improve our ability to identify those histopathological DCIS features that matter the most in terms of iIBC risk, on which future studies which aim to optimize reliability should focus.

In univariable analysis, patients treated with radiotherapy after BCS had a strongly reduced risk of iIBC compared to those treated by BCS alone, as was already shown previously [30, 36, 37]. Also grade 3 (versus grade 1 + 2 combined), a high mitotic activity and a cribriform/solid growth pattern (versus FEA, clinging, or (micro)papillary growth pattern) were associated with increased iIBC risk in patients treated with BCS alone. In multivariable analysis however, only grade 3 (versus grade 1 + 2) and a cribriform/solid growth pattern were independently associated with an increased iIBC risk. Mitotic activity did not add any predictive value to grade 3 versus 1 + 2 and growth pattern in a multivariable model, though this is likely due to collinearity with grade. Another important finding in our study is that no histopathological features were associated with iIBC risk in the patients treated with BCS + RT. Although women in our study were not randomized for treatment arm, this finding may suggest that radiotherapy neutralizes the effect of these classical histopathological features. This is also in line with the fact that within the large randomized controlled trials of RT in DCIS no subgroup could be identified without RT benefit [36].

So far, grade is the sole histopathological feature in DCIS that is used in clinical practice and also has an impact on eligibility in the context of clinical trials investigating the safety of active surveillance in low-risk DCIS [38–40]. In general, only women over the age of 45 or 50 with screen-detected calcifications associated with DCIS grade 1 or grade 2 are eligible in these trials. A three-tiered grading system is used for this selection purpose. Our study supports the rationale to distinguish between grade 1 + 2 versus grade 3 as DCIS grade 3 is independently associated with an increased risk of iIBC in patients treated with BCS alone. Unfortunately, the interrater reliability of assessing grade using either a 3-tiered grading system (grade 1, 2, or 3) or a 2-tiered system differentiating grade 1 + 2 combined versus grade 3 was only fair when considering KA and at best moderate based on the GAC2.

The interrater reliability for growth pattern was moderate (KA) to substantial (GAC2). The predictive ability of grade and growth pattern has been intensively studied previously, with conflicting results [10]. Factors such as substantial interrater variability, grading system used, bias in designs, and relying on histopathological assessments of a single pathologist’s opinion may have resulted in these different findings [10]. Interrater reliability based on GAC2 was higher overall, when histopathological features showed strongly skewed distribution and when agreement was already very high (i.e., grade 1 versus 2 + 3, growth pattern, and mitotic activity). Under these circumstances, a GAC2 test may result in more accurate reliability coefficients, as was previously shown in comparison with Cohen’s kappa, which overestimates the concordance attributed due to chance alone in these situations leading to lower reliability coefficients [41].

In view of the prognostic value and interrater reliability observed in our study, it is questionable whether it is safe to base clinical treatment decisions solely on the assessment of classical histopathological features. Here, we propose four strategies that may improve risk stratification in DCIS.

Within the context of DCIS, the three features with reasonable prognostic value (grade 1 + 2 versus 3, growth pattern, and mitotic activity) are currently used in many grading systems, but without clear definitions and rules about how to value each feature. We therefore firstly would suggest to objectify histological grading by using a numerical semi-quantitative scoring system which separately evaluates each of these features, analogous to the modified Bloom and Richardson grading system for IBC [42, 43]. Dichotomous scoring systems may further improve reliability and prognostic value and should be further explored evaluating different cut-offs [44, 45].

Secondly, performing additional immunohistochemistry to assign specific DCIS profiles may add prognostic value, possibly only in subsets of patients (i.e., grade 2). Previously, associations were reported of human epidermal growth factor receptor 2 (HER2)-positive, estrogen receptor (ER)-negative DCIS, and DCIS with high cyclooxygenase 2, p16, and Ki-67 levels with increased iIBC risk [9, 10, 46, 47]. These markers would be good candidates for further exploration. Automated scoring within this context may result in more standardized and objective assessment [48–51]. Previously, a 3-tiered grading system in DCIS, combining nuclear grade according to the Van Nuys criteria with automated Ki-67 count, was reported to show excellent correlation with immunohistochemical markers of reported biological relevance such as ER and HER2 [9, 46, 47, 50].

Thirdly, alternative approaches using pathology information such as artificial intelligence-based methods should also be considered in search for clinically relevant biomarkers in DCIS [52]. Recently, others have developed a whole slide image-based machine learning model, which accurately predicted the risk of an invasive or in situ recurrence and significantly outperformed traditional clinicopathological variables [53].

Lastly, besides pathology, other criteria could also be incorporated in clinical decision schemes, e.g., as in current active surveillance trials requiring DCIS to be screen-detected based on calcifications only without clinical symptoms and diagnosed on representative vacuum-assisted biopsies [38–40].

Our study had several limitations. From our study population, each rater scored a different subset of patients. Therefore, we were not able to analyze the association of histopathological DCIS features with iIBC risk per rater or grading system used and to study the effect of interrater variability on risk stratification. However, the resulting immense workload would probably have caused major rater-dropout. Also tissue slides were digitally assessed using research technology producing images of somewhat lower resolution. This may have led to difficulty of assessing histopathological features requiring great detail, such as mitotic activity. Our reliability study was nonetheless performed under conditions as close as possible to clinical practice, as a large set of non-selected DCIS cases from a population-based cohort were reviewed by a large group of raters with varying levels of expertise without provision of instructions or tutorials beforehand. And lastly, data on margin status and DCIS lesion size, factors potentially associated with the risk of iIBC, were not collected in a standardized way [10, 46, 47, 54]. However, Dutch guidelines state that a re-excision or mastectomy is obligatory in case of involved margins after a primary excision. An explorative analysis using the available data on margin status indeed showed no significant difference in the risk of iIBC for positive margins and even a protective effect for close margins in women treated with BCS alone in comparison to women with negative margins, suggesting they were subjected to re-excisions.

## Conclusions

We evaluated the prognostic value of histopathological DCIS features to inform risk stratification using a unique, combined approach. Our study showed substantial interrater variability in the classification of histopathological DCIS features, while using rater majority opinions, minimizing the muddling effect of interrater variability, DCIS grade, growth pattern, and mitotic activity were associated with the risk of subsequent ipsilateral invasive breast cancer after DCIS in patients treated with BCS without radiotherapy. A semi-quantitative grading system incorporating and separately evaluating nuclear pleomorphism, growth pattern, and mitotic activity, analogue to IBC grading, may improve the reliability and prognostic value of these histopathological features.

## Electronic supplementary material

Below is the link to the electronic supplementary material.

Supplementary file1 (DOCX 63 kb)

## Data Availability

The datasets generated and/or analyzed during the current study are available from the corresponding author upon reasonable request. Requests should be made to Prof. J. Wesseling: j.wesseling@nki.nl.

## Abbreviations

DCIS: Ductal carcinoma in situ
IBC: Invasive breast cancer
iIBC: Ipsilateral invasive breast cancer
95% CI: 95% Confidence Interval
BCS ± RT: Breast-conserving surgery with or without radiotherapy
NCR: Netherlands Cancer Registry
PALGA: The nationwide network and registry of histology and cytopathology in the Netherlands.
KA: Krippendorff’s alpha
GAC2: Gwet’s AC2
PHA: Proportional hazard assumptions
HR: Hazard Ratio
FEA: Flat epithelial atypia
HER2: Human epidermal growth factor receptor 2
ER: Estrogen receptor
