# Supplementary material for: Prognostic value of histopathological DCIS features in a large-scale international interrater reliability study
Source: Breast Cancer Res Treat. 2020 Jul 30;183(3):759–70. doi: 10.1007/s10549-020-05816-x (PMC7497690; doi:10.1007/s10549-020-05816-x)
Supplement: Supplementary file 1 — Supplementary file1 (DOCX 63 kb) [file 10549_2020_5816_MOESM1_ESM.docx]

**Supplementary methods**

**Rater selection and participation**

To ensure a mixed group of raters in terms of expertise and experience, a dual selection approach was undertaken. Members of the European Working Group for Breast Screening Pathology, a working group set up in 1993 in order to make the practice of breast pathology more uniform and considered breast pathology experts, were invited to participate by email. Twenty-two members agreed to participate and 17 completed the study.

All participants of the ‘7^th^ Dutch Breast Pathology Course’ (November 2018, Amsterdam; 31 pathologists and 3 residents) with different levels of expertise were also invited to participate in the study. Nineteen pathologists and 2 residents completed the study, for which the first received CME accreditation as compensation.

After study-closure all raters who completed the study received personal feedback by providing an overview comparing their scores with those from the group.

**Study sets**

To reduce the workload while ensuring enough ratings per case for subsequent analysis, each rater was assigned a personal study set, including in total 146 cases. The study sets were composed in two steps. Firstly, 100 cases were randomly selected from the total cohort of 353 cases and assigned to the study sets of all raters. Secondly, for each rater individually 46 cases out of the remaining 253 cases not yet assigned, were randomly selected and added to their study set.

Fifty out of the 100 cases, which were assigned to all raters, were placed in the beginning of the study set and the other fifty were randomly distributed amongst the remaining cases. Raters were aware of a presumed DCIS diagnosis in this study and were not restricted in scoring time (starting date 15/10/2018 - closing date 08/02/2019).

**DCIS scoring form**

1. DCIS present? (if not, please give the diagnosis under comments)
   - Yes
   - No
   - Not assessable
2. Dominant growth pattern?
   - Not assessable
   - FEA
   - Clinging
   - (Micro)papillary
   - Cribriform
   - Solid
3. DCIS grade? (1/2/3)
   - Not assessable
   - Well differentiated
   - Moderately differentiated
   - Poorly differentiated
4. DCIS grade? (low/high)
   - Not assessable
   - Low grade
   - High grade
5. Necrosis present?
   - Not assessable
   - Absent
   - Present
6. Calcification present?
   - Not assessable
   - Absent
   - Present
7. Frequency of mitoses?
   - Not assessable
   - Sparse
   - Many
8. Periductal fibrosis present?
   - Not assessable
   - Absent
   - Subtle
   - Prominent
9. Only if fibrosis is present: what is the (dominant) type of stroma?
   - Not assessable
   - Sclerotic
   - Myxoid
10. Lymphocytic infiltrate present?
    - Not assessable
    - Absent
    - Subtle
    - Prominent

Comments (other diagnosis or otherwise)

1 = well differentiated/2 = moderately differentiated/3 = poorly differentiated; FEA = flat epithelial atypia

**DCIS Interobserver Study – rater background questionnaire**

1. Your email address ^a^
2. In which country are you working?
3. In which hospital/pathology lab are you working?
4. Where did you receive your pathology training? (hospital/place/country)
5. How many years are you working as a pathologist?

0-5 years

6-10 years

11-15 years

16-20 years

>20 years

6. How many years are you looking at breast cases?

0-5 years

6-10 years

11-15 years

16-20 years

>20 years

7. Do your colleagues consider you an expert in breast pathology?

Yes

No

8. How many pathologists are working in your lab?

9. How many pathologists are looking at breast cases in your lab?

10. How many breast cases are seen annually in your lab (estimate, biopsies + surgical specimens)

11. Do you look at revision or consult cases?

Yes

No

12. Which DCIS grading system do you use in daily practice?

Holland et al (1994; 3-tiered; based on nuclear grade and cell polarization)

Pinder et al (2010; 4-tiered; very high = high nuclear grade + >50% solid growth & comedo-necrosis)

Van Nuys (1995; 3-tiered; high grade, non-high grade with necrosis, non-high grade without necrosis)

Poller et al. (1994; 2-tiered; pure comedo, non comedo)

Lagios (1990; 3-tiered; based on nuclear features & frequency of mitoses)

College of American Pathologists Guidelines

WHO

Intuition

Other:

13. In case of a heterogeneous DCIS, how did you grade in this study?

I gave the highest grade

I gave the predominant grade

Other:

14. Comments regarding your interpretation of specific items in the study

15. How would you rate the slide viewing platform ‘Slide Score’?

16. Comments/feedback for Slide Score

A questionnaire was sent to all 38 raters who finished their complete study set with questions regarding their working environment, experience and their method of DCIS grading. Thirty-five pathologists and 2 residents completed the questionnaire.

^a^ Required

| **Supplementary Table S1. Clinical characteristics of included and excluded patients for iIBC risk analysis** | | | | | |  |
| --- | --- | --- | --- | --- | --- | --- |
|  | **Subcohort patients** | |  | **Patients outside subcohort with subsequent iIBC** | |  |
|  | **Included patients n (%) 215 (60.2)** | **Excluded patients n (%) 142 (39.8)** | ***P^a^*** | **Included patients n (%) 117 (66.1)** | **Excluded patients n (%) 60 (33.9)** | ***P^a^*** |
| **Patient group** |  |  |  |  |  |  |
| Subcohort, no iIBC | 195 (90.7) | 131 (92.3) |  |  |  |  |
| Subcohort, iIBC | 20 (9.3) | 11 (7.8) | 0.61 |  |  |  |
| **Treatment** |  |  |  |  |  |  |
| BCS+RT | 128 (59.5) | 77 (54.2) |  | 34 (29.1) | 24 (40.0) |  |
| BCS alone | 87 (40.5) | 65 (45.8) | 0.32 | 83 (70.9) | 36 (60.0) | 0.14 |
| **Age at DCIS diagnosis, years, median (iqr)** | 58.4 (53.4-64.0) | 58.3 (53.3-64.2) | 0.68 | 57.5 (53.2-63.6) | 59.0 (54.5-62.0) | 0.63 |
| **Age at DCIS diagnosis, years**  **(quartiles)** |  |  |  |  |  |  |
| ≥49.5 - ≤53.4 | 54 (25.1) | 38 (26.8) |  | 30 (25.6) | 13 (21.7) |  |
| >53.4 - ≤58.3 | 53 (24.7) | 33 (23.2) |  | 32 (27.4) | 15 (25.0) |  |
| >58.3 - ≤63.7 | 53 (24.7) | 31 (21.8) |  | 27 (23.1) | 23 (38.3) |  |
| >63.7 - ≤75.6 | 55 (25.6) | 40 (28.2) | 0.88 | 28 (23.9) | 9 (15.0) | 0.16 |
| **Period of DCIS diagnosis**^b^ |  |  |  |  |  |  |
| 1993 - 1998 | 82 (38.1) | 58 (40.9) |  | 63 (53.9) | 43 (71.7) |  |
| 1999 - 2004 | 133 (61.9) | 84 (59.2) | 0.61 | 54 (46.2) | 17 (28.3) | **0.022** |

Subcohort = randomly selected patient group; n = number; *P* = P value; ^a^ For categorical variables the P value was calculated by a chi-square test, for age at diagnosis by a Wilcoxon rank-sum test; iIBC = ipsilateral invasive breast cancer; iqr = interquartile range; ^b^ 1993-1998 reflecting part of the screening implementation phase and 1999-2004 reflecting full nationwide coverage

| **Supplementary Table S2. Number of scores per slide and agreement with the majority opinion per histopathological feature** | | | | |  |
| --- | --- | --- | --- | --- | --- |
|  | **n of scores per slide** | | **Agreement with the majority opinion score (%)** | |  |
| **Histopathological feature** | **Mean** | **Median (iqr)** | **Mean** | **Median (iqr)** |  |
| **Grade (1,2 or 3)** | 14 | 7 (6-32) | 70.1 | 69.4 (57.1-83.3) |  |
| **Grade (1 versus 2+3)** | 14 | 7 (6-32) | 89.8 | 97.3 (83.3-100) |  |
| **Grade (1+2 versus 3)** | 14 | 7 (6-32) | 79.4 | 83.3 (66.7-100) |  |
| **Grade (low versus high)** | 14 | 7 (6-30) | 83.0 | 85.3 (71.4-100) |  |
| **Dominant growth pattern**^a^ | 15 | 7 (6-32) | 90.4 | 100 (83.3-100) |  |
| **Calcifications** | 15 | 7 (6-32) | 88.2 | 97.1 (80.0-100) |  |
| **Necrosis** | 15 | 7 (6-33) | 88.2 | 95.4 (80.0-100) |  |
| **Mitotic activity** | 13 | 7 (6-29) | 86.4 | 93.8 (75.0-100) |  |
| **Periductal fibrosis (absent, subtle or prominent presence)** | 15 | 7 (6-32) | 65.1 | 62.5 (54.1-75.0) |  |
| **Periductal fibrosis**  **(present versus absent)** | 15 | 7 (6-32) | 81.6 | 83.3 (71.4-100) |  |
| **Type of periductal fibrosis**^b^ | 12 | 6 (5-24) | 81.7 | 83.3 (66.7-100) |  |
| **Lymphocytic infiltrate (absent, subtle or prominent presence)** | 15 | 7 (6-31) | 71.1 | 67.6 (57.1-83.3) |  |
| **Lymphocytic infiltrate**  **(present versus absent)** | 15 | 7 (6-31) | 82.4 | 83.8 (66.7-100) |  |

n = number; iqr = interquartile range; ^a^ in one patient growth pattern was scored as not assessable by all raters and was therefore excluded (n included patients = 341); ^b^ for type of fibrosis patients were only included when according to the majority opinion periductal fibrosis was present, either subtle or prominent (n included patients = 276)

| **Supplementary Table S3. Characteristics of raters participating in the study^a,b^** | |
| --- | --- |
| **Experience, years** | **n (%)** |
| 0-5 | 5 (15.2) |
| 6-10 | 2 (6.1) |
| 11-15 | 3 (9.1) |
| 16-20 | 5 (15.2) |
| > 20 | 18 (54.6) |
| **Country of work** |  |
| the Netherlands | 17 (48.6) |
| Europe, other | 18 (51.4) |
| **EWGBSP-member** |  |
| Yes | 17 (47.2) |
| No | 19 (52.8) |
| **Considered expert in breast pathology by colleagues** |  |
| Yes | 30 (88.2) |
| No | 4 (11.8) |
| **Experience with breast revision/consult cases** |  |
| Yes | 26 (74.3) |
| No | 9 (25.7) |
| **DCIS grading system used** |  |
| WHO[1] | 9 (25.0) |
| Holland[2] | 10 (27.8) |
| Van Nuys[3] | 4 (11.1) |
| WHO & Van Nuys | 4 (11.1) |
| WHO & Holland | 2 (5.6) |
| WHO & Holland & Lagios[4] | 1 (2.8) |
| WHO & CAP[5] | 1 (2.8) |
| Lagios | 1 (2.8) |
| Pinder[6] | 1 (2.8) |
| Other | 3 (8.3) |
| **Grading in case of heterogeneous DCIS** |  |
| Highest grade | 33 (94.3) |
| Predominant grade | 2 (5.7) |
| **Supplementary Table S3 continued.** |  |
| **Characteristics of the raters’ laboratories**  **n of pathologists, median (iqr)** | 13 (8-15) |
| **n of breast pathologists, median (iqr)** | 4 (3-5) |
| **Laboratory specialization^c^, median (iqr)** | 2.6 (1.8-4.6) |
| **n of breast cases seen annually, median (iqr)** | 1200 (600-2000) |
|  |  |
| ^a^ the questionnaire was not filled in (completely) by all raters, percentages are based on the responders; ^b^ Residents are included only in questions regarding their grading of DCIS  n = number; iqr = interquartile range; EWGBSP = members of the European Working Group for Breast Screening Pathology; ^c^ Laboratory specialization = number of pathologists in rater’s laboratory/number of breast pathologists in rater’s laboratory | |
|  | |

| **Supplementary Table S4. Associations of clinicopathological characteristics with subsequent iIBC in univariable analysis** | | | | | | | | | | |
| --- | --- | --- | --- | --- | --- | --- | --- | --- | --- | --- |
| **Clinicopathological characteristic** | **All patients** | | | **BCS alone** | | | **BCS+RT** | | | **Interaction** |
|  | **n** | **HR (95% CI)** | ***P*** | **n** | **HR (95% CI)** | ***P*** | **n** | **HR (95% CI)** | ***P*** | ***P*** |
| **Grade (1,2 or 3)^a^** |  |  |  |  |  |  |  |  |  |  |
| 1 | 31 (10) | **REF** |  | 21 (8) | **REF** |  | 10 (2) | **REF** |  |  |
| 2 | 172 (67) | 1.28 (0.58-2.83) | 0.54 | 84 (43) | 1.61 (0.63-4.08) | 0.32 | 88 (24) | 1.39 (0.27-7.15) | 0.69 | 0.94 |
| 3 | 129 (60) | 1.69 (0.75-3.80) | 0.20 | 65 (44) | 3.19 (1.21-8.37) | **0.019** | 64 (16) | 1.10 (0.20-5.89) | 0.91 | 0.33 |
| **Grade (1 versus 2+3)** |  |  |  |  |  |  |  |  |  |  |
| 1 | 30 (10) | **REF** |  | 21 (8) | **REF** |  | 9 (2) | **REF** |  |  |
| 2+3 | 302 (127) | 1.35 (0.62-2.91) | 0.45 | 149 (87) | 2.15 (0.88-5.22) | 0.092 | 153 (40) | 0.98 (0.19-5.14) | 0.99 | 0.50 |
| **Grade (1+2 versus 3)** |  |  |  |  |  |  |  |  |  |  |
| 1+2 | 211 (80) | **REF** |  | 107 (52) | **REF** |  | 104 (28) | **REF** |  |  |
| 3 | 121 (57) | 1.41 (0.90-2.20) | 0.13 | 63 (43) | 2.34 (1.24-4.42) | **0.009** | 58 (14) | 0.74 (0.35-1.56) | 0.42 | **0.028** |
| **Grade (low versus high)** |  |  |  |  |  |  |  |  |  |  |
| Low | 87 (31) | **REF** |  | 54 (27) | **REF** |  | 33 (4) | **REF** |  |  |
| High | 245 (106) | 1.33 (0.81-2.20) | 0.26 | 116 (68) | 1.47 (0.79-2.76) | 0.23 | 129 (38) | 2.68 (0.88-8.21) | 0.084 | 0.34 |
| **Dominant growth pattern^b^** |  |  |  |  |  |  |  |  |  |  |
| FEA/clinging/(micro)papillary | 46 (14) | **REF** |  | 23 (7) |  |  | 23 (7) | **REF** |  |  |
| Cribriform/solid | 285 (123) | 1.76 (0.92-3.36) | 0.087 | 146 (88) | 3.44 (1.33-8.91) | **0.011** | 139 (35) | 0.70 (0.29-1.72) | 0.44 | **0.023** |
|  | | | | | | | | | | |
| **Supplementary Table S4 continued.** | | | | | | | | | | |
| **Clinicopathological characteristic** | **All patients** | |  | **BCS alone** | |  | **BCS+RT** |  |  | **Interaction** |
|  | **n** | **HR (95% CI)** | ***P*** | **n** | **HR (95% CI)** | ***P*** | **n** | **HR (95% CI)** | ***P*** | ***P*** |
| **Calcifications** |  |  |  |  |  |  |  |  |  |  |
| Present | 256 (103) | **REF** |  | 131 (71) | **REF** |  | 125 (32) | **REF** |  |  |
| Absent | 76 (34) | 1.23 (0.75-2.04) | 0.41 | 39 (24) | 1.31 (0.65-2.65) | 0.45 | 37 (10) | 1.13 (0.51-2.53) | 0.77 | 0.76 |
| **Necrosis** |  |  |  |  |  |  |  |  |  |  |
| Present | 260 (109) | **REF** |  | 126 (72) | **REF** |  | 134 (37) | **REF** |  |  |
| Absent | 72 (28) | 0.87 (0.52-1.46) | 0.59 | 44 (23) | 0.80 (0.41-1.56) | 0.51 | 28 (5) | 0.60 (0.22-1.65) | 0.32 | 0.59 |
| **Mitotic activity** |  |  |  |  |  |  |  |  |  |  |
| Sparse | 294 (114) | **REF** |  | 141 (74) | **REF** |  | 153 (40) | **REF** |  |  |
| Many | 38 (23) | 2.42 (1.20-4.91) | **0.014** | 29 (21) | 2.53 (1.05-6.11) | **0.038** | 9 (2) | 0.79 (0.15-4.15) | 0.78 | 0.21 |
| **Periductal fibrosis^a^** |  |  |  |  |  |  |  |  |  |  |
| Absent | 64 (28) | **REF** |  | 42 (24) | **REF** |  | 22 (4) | **REF** |  |  |
| Subtle | 165 (73) | 1.02 (0.58-1.78) | 0.95 | 84 (48) | 1.01 (0.50-2.05) | 0.98 | 81 (25) | 1.98 (0.63-6.20) | 0.24 | 0.33 |
| Prominent | 103 (36) | 0.70 (0.38-1.31) | 0.27 | 44 (23) | 0.84 (0.36-1.91) | 0.67 | 59 (13) | 1.29 (0.39-4.30) | 0.68 | 0.56 |
| **Periductal fibrosis present/ absent** |  |  |  |  |  |  |  |  |  |  |
| Present (subtle/prominent) | 275 (113) | **REF** |  | 134 (75) | **REF** |  | 141 (38) | **REF** |  |  |
| Absent | 57 (24) | 1.06 (0.61-1.84) | 0.84 | 36 (20) | 0.97 (0.48-1.96) | 0.94 | 21 (4) | 0.67 (0.22-2.02) | 0.48 | 0.56 |
|  | | |  |  | | |  |  |  |  |
| **Supplementary Table S4 continued.** | | |  |  | | |  |  |  |  |
| **Clinicopathological characteristic** | **All patients** | |  | **BCS alone** | | | **BCS+RT** |  |  | **Interaction** |
|  | **n** | **HR (95% CI)** | ***P*** | **n** | **HR (95% CI)** | ***P*** | **n** | **HR (95% CI)** | ***P*** | ***P*** |
| **Type of periductal fibrosis^c^** |  |  |  |  |  |  |  |  |  |  |
| Sclerotic | 202 (80) | **REF** |  | 101 (54) | **REF** |  | 101 (26) | **REF** |  |  |
| Myxoid | 66 (29) | 1.29 (0.74-2.24) | 0.37 | 27 (17) | 2.23 (0.86-5.76) | 0.099 | 39 (12) | 1.18 (0.53-2.62) | 0.68 | 0.34 |
| **Lymphocytic infiltrate^a^** |  |  |  |  |  |  |  |  |  |  |
| Absent | 108 (38) | **REF** |  | 58 (30) | **REF** |  | 50 (8) | **REF** |  |  |
| Subtle | 144 (65) | 1.48 (0.90-2.44) | 0.12 | 77 (42) | 1.11 (0.58-2.14) | 0.75 | 67 (23) | 2.74 (1.12-6.69) | **0.027** | 0.11 |
| Prominent | 80 (34) | 1.35 (0.75-2.41) | 0.32 | 35 (23) | 1.91 (0.80-4.54) | 0.14 | 45 (11) | 1.56 (0.58-4.21) | 0.38 | 0.79 |
| **Lymphocytic infiltrate present/absent** |  |  |  |  |  |  |  |  |  |  |
| Present (subtle/prominent) | 227 (100) | **REF** |  | 113 (66) | **REF** |  | 114 (34) | **REF** |  |  |
| Absent | 105 (37) | 0.71 (0.45-1.13) | 0.15 | 57 (29) | 0.73 (0.39-1.35) | 0.31 | 48 (8) | 0.50 (0.22-1.16) | 0.11 | 0.46 |
| **Age at diagnosis, years (quartiles)** |  |  |  |  |  |  |  |  |  |  |
| ≥49.5 - ≤53.4 | 84 (37) | **REF** |  | 38 (20) | **REF** |  | 46 (17) | **REF** |  |  |
| >53.4 - ≤58.2 | 82 (36) | 0.97 (0.53-1.76) | 0.92 | 43 (24) | 1.12 (0.47-2.64) | 0.80 | 39 (12) | 0.73 (0.30-1.79) | 0.49 | 0.51 |
| >58.2 - ≤63.8 | 83 (32) | 0.81 (0.44-1.48) | 0.49 | 43 (26) | 1.24 (0.53-2.90) | 0.61 | 40 (6) | 0.33 (0.11-0.92) | **0.035** | **0.048** |
| >63.8 - ≤75.6 | 83 (32) | 0.84 (0.46-1.53) | 0.57 | 46 (25) | 1.02 (0.45-2.34) | 0.96 | 37 (7) | 0.46 (0.17-1.26) | 0.13 | 0.20 |
| **Supplementary Table S4 continued.** | | | | | | | | | | |
| **Clinicopathological characteristic** | **All patients** | |  | **BCS alone** | |  | **BCS+RT** |  |  | **Interaction** |
|  | **n** | **HR (95% CI)** | ***P*** | **n** | **HR (95% CI)** | ***P*** | **n** | **HR (95% CI)** | ***P*** | ***P*** |
| **Age at diagnosis (cont.)** |  | 0.98 (0.95-1.02) | 0.38 |  | 1.00 (0.96-1.05) | 0.90 |  | 0.93 (0.86-1.00) | 0.053 | 0.079 |
| **Period of DCIS diagnosis** |  |  |  |  |  |  |  |  |  |  |
| 1993 - 1998 | 145 (76) | **REF** |  | 104 (63) | **REF** |  | 41 (13) | **REF** |  |  |
| 1999 - 2004 | 187 (61) | 0.61 (0.39-0.96) | **0.032** | 66 (32) | 0.75 (0.41-1.37) | 0.35 | 121 (29) | 1.44 (0.58-3.57) | 0.44 | 0.66 |
| **Treatment** |  |  |  |  |  |  |  |  |  |  |
| BCS+RT / 0-5 years | 162 (14) | **REF** |  |  |  |  |  |  |  |  |
| BCS+RT / >5 years | 142 (28) | 0.51 (0.24-1.12) | 0.093 |  |  |  |  |  |  |  |
| BCS alone / 0-5 years | 170 (43) | 4.80 (2.49-9.24) | **0.000** |  |  |  |  |  |  |  |
| BCS alone / >5 years | 118 (52) | 2.47 (1.42-4.30) | **0.001** |  |  |  |  |  |  |  |
|  |  | ***P*_heterogeneity_** | **0.000** |  |  |  |  |  |  |  |

n = total number (number of patients with subsequent iIBC); HR = Hazard Ratio; CI = Confidence Interval; *P* = P value; Interaction = interaction with treatment; REF = reference; cont. = Continuous; ^a^ Recategorizations of grade, periductal fibrosis, and lymphocytic infiltrate may have led to small differences in the majority opinion (for example when considering the histopathological feature grade 1-3 with a distribution of grade 1 -30%, grade 2 -30% and grade 3 -40% with grade 3 as majority opinion will lead to a categorical shift when recategorizing grade 1-3 into grade 1+2 versus 3 with an adjusted distribution of grade 1 or 2 - 60% and grade 3 -40% with grade 1+2 as majority opinion); ^b^ in one patient growth pattern was scored as not assessable by all raters and was therefore excluded (n included patients = 331); ^c^ for type of fibrosis patients were only included when according to the majority opinion periductal fibrosis was present, either subtle or prominent (n included patients = 268)

**References**

1. Lakhani SR, Ellis. I.O., Schnitt SJ, Tan PH, van de Vijver MJ. WHO classification of tumours of the breast. 4th ed. Lyon: International Agency for Research on Cancer; 2012.

2. Holland R, Peterse JL, Millis RR, Eusebi V, Faverly D, Van de Vijver MJ, et al. Ductal carcinoma in situ: A proposal for a new classification. Semin Diagn Pathol. 1994;11(3):167–80.

3. Silverstein MJ, Poller DN, Waisman JR, Colburn WJ, Barth A, Gierson ED, et al. Prognostic classification of breast ductal carcinoma-in-situ. Lancet. 1995;345(8958):1154–7.

4. Lagios MD. Duct carcinoma in situ. Pathology and treatment. Surg Clin North Am. 1990;70(4):873–83.

5. College of American pathologists [Internet]. Available from: https://documents.cap.org/protocols/cp-breast-dcis-18protocol-4100.pdf

6. Pinder SE, Duggan C, Ellis IO, Cuzick J, Forbes JF, Bishop H, et al. A new pathological system for grading DCIS with improved prediction of local recurrence: Results from the UKCCCR/ANZ DCIS trial. Br J Cancer. 2010;
